# Characterization of the role of autophagy in retinal ganglion cell survival over time using a rat model of chronic ocular hypertension

**DOI:** 10.1038/s41598-021-85181-x

**Published:** 2021-03-11

**Authors:** Si Hyung Lee, Kyung Sun Shim, Chan Yun Kim, Tae Kwann Park

**Affiliations:** 1 Department of Ophthalmology, College of Medicine, Soonchunhyang University, Cheonan, Republic of Korea; 2 Department of Ophthalmology, Soonchunhyang University Hospital Bucheon, 170 Jomaru-ro, Wonmi-gu, Bucheon, 14584 Republic of Korea; 3 Institute of Vision Research, Department of Ophthalmology, College of Medicine, Severance Hospital, Yonsei University, Seoul, South Korea

**Keywords:** Preclinical research, Glaucoma, Autophagy, Cell death

## Abstract

Autophagy is an essential cellular process for the degradation and recycling of cellular components, and its dysregulation has been linked to neuronal cell death and neurodegeneration. In glaucoma, the role of autophagy in retinal ganglion cell (RGC) survival remains contradictory. Moreover, the effects of autophagy modulation at different time-points on RGC survival in a glaucoma model have not been investigated. In this study, we assessed the time-dependent role of autophagy in RGC survival in a circumlimbal suture-induced ocular hypertensive (OHT) rat model. Intraocular pressure (IOP) elevation led to a gradual autophagy induction, which reached a maximum between 1 and 4 weeks after OHT induction. On the other hand, early autophagy was impaired between 1 and 3 days after circumlimbal suturing, indicated by increased p62 levels due to reduced autophagosomal turnover. The intravitreal administration of rapamycin at different time-points after the application of the circumlimbal suture indicated that autophagy induction early during OHT development had potent survival-promoting effects in RGCs. In conclusion, our findings suggest that the role of autophagy in RGCs during OHT development might differ in a time-dependent manner. Modulating autophagy at the appropriate time might serve as a potential therapeutic approach to enhance RGC survival in OHT.

## Introduction

Glaucoma is a leading cause of irreversible blindness worldwide. It is a chronic progressive glaucomatous optic neuropathy involving the gradual loss of retinal ganglion cells (RGCs), resulting in visual-field defects^1,2^. Increased intraocular pressure (IOP) is an important risk factor for glaucoma, as it often compresses the optic nerve at the lamina cribrosa, impeding retrograde axonal transportation and causing RGC apoptosis. Current treatment for glaucoma mainly involves interventions that lower the IOP and prevent glaucomatous damage. However, many ongoing studies aim to identify mechanisms to promote RGC survival in patients with glaucoma. Therefore, the elucidation of the mechanisms underlying RGC death under high IOP conditions is key in developing effective neuroprotective therapies.

Autophagy is a cellular process essential for the physiological turnover of dysfunctional organelles, proteins, and long-lived misfolded proteins^3^. Macroautophagy, one of the three main autophagy mechanisms, involves the formation of autophagosomes, engulfing cytosolic components^4,5^. Macroautophagy is vital for cell adaptation to stressful conditions, allowing them to survive under nutrient-deprived conditions^6^. Nevertheless, overactivation of autophagy might be detrimental for cell survival, leading to self-digestion and cellular apoptosis^7^.

Under physiological conditions, autophagy occurs in neurons at low levels. Numerous studies have suggested that autophagy inhibition led to neurodegeneration in various experimental models^8–13^. Mounting evidence from in vitro and in vivo studies suggests that in ocular neurodegenerative diseases, including glaucoma, autophagy has neuroprotective effects on RGCs^14–16^. On the other hand, some studies have shown that autophagy might have unfavorable effects in patients with glaucomatous conditions, as it might promote retinal neuronal cell death^17–19^. However, considering that the autophagic flux might differ according to the time after an unfavorable insult, it is necessary to establish the ideal time to activate autophagy to ensure its neuroprotective effects. To this end, we investigated the time-dependent changes in the autophagic flux and its underlying molecular mechanisms in an ocular hypertensive (OHT) animal model. Moreover, using rapamycin to induce autophagy, we established the most appropriate time to activate autophagy to enhance its survival-promoting effects on RGCs under OHT condition.

## Results

### Establishment of a chronic ocular hypertensive rat model by circumlimbal suture placement

**Figure 1:**
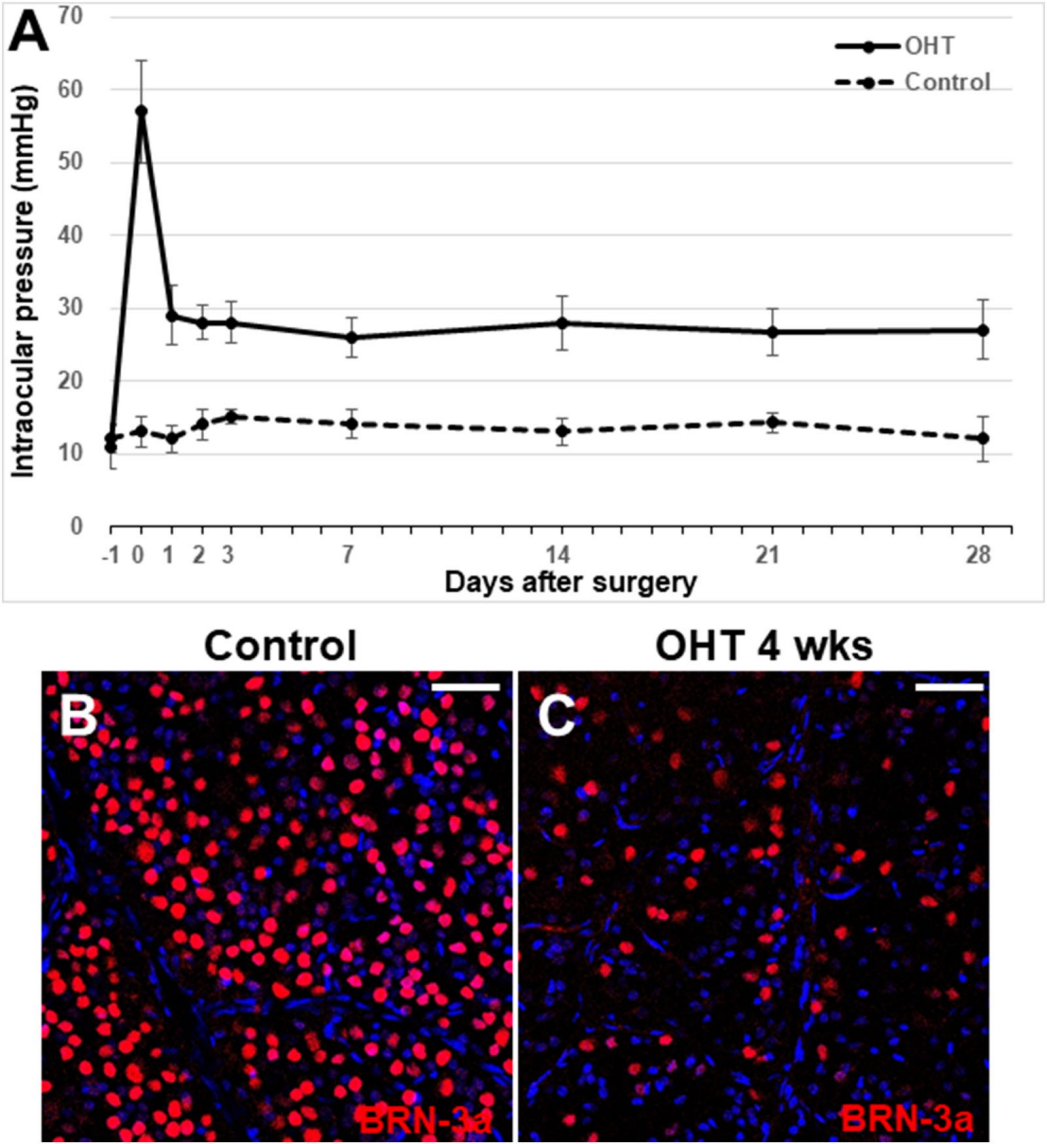
Establishment of a rat model of OHT by circumlimbal suture placement. (**A**) Immediately after circumlimbal suturing, IOP was significantly elevated compared to the baseline IOP (57 ± 7 mmHg), which then decreased and remained lower until 4 weeks of the suturing (27 ± 4 mmHg). This was significantly higher compared to the IOP in control eyes (12 ± 3 mmHg, p < 0.001). (**B**,**C**) Retinal whole-mount immunostaining with BRN-3a revealed a profound decrease in the number of RGCs in retinas from OHT rats (**B**) compared to the control rats (**C**). Representative images (**B**,**C**) were taken from middle (2 mm from the optic disc) regions of the retinal whole mounts. Data are presented as means ± SEsM. OHT, ocular hypertension. Scale bar, 40 µm.

We employed a circumlimbal suture technique to develop an OHT rat model, which has previously been described^20,21^. Baseline IOPs for the animals of the OHT group and control group were 11 ± 3 mmHg and 10 ± 4 mmHg, respectively. Immediately after the surgery, IOP increased significantly (57 ± 7 mmHg) from the baseline IOP and then decreased to 29 ± 4 mmHg 1 day after the circumlimbal suture placement. One month after circumlimbal suturing, IOP in OHT eyes was still significantly higher (27 ± 4 mmHg) compared to that in control eyes (12 ± 3 mmHg, p < 0.001) (Fig. 1A). Furthermore, we found a profound decrease in the number of RGCs expressing BRN-3a in retinal whole-mounts of OHT eyes compared to the retinas from control animals, confirming the development of OHT eyes (Fig. 1B,C).

### Time-dependent changes in LC3B and p-mTOR expression levels in OHT eyes

**Figure 2:**
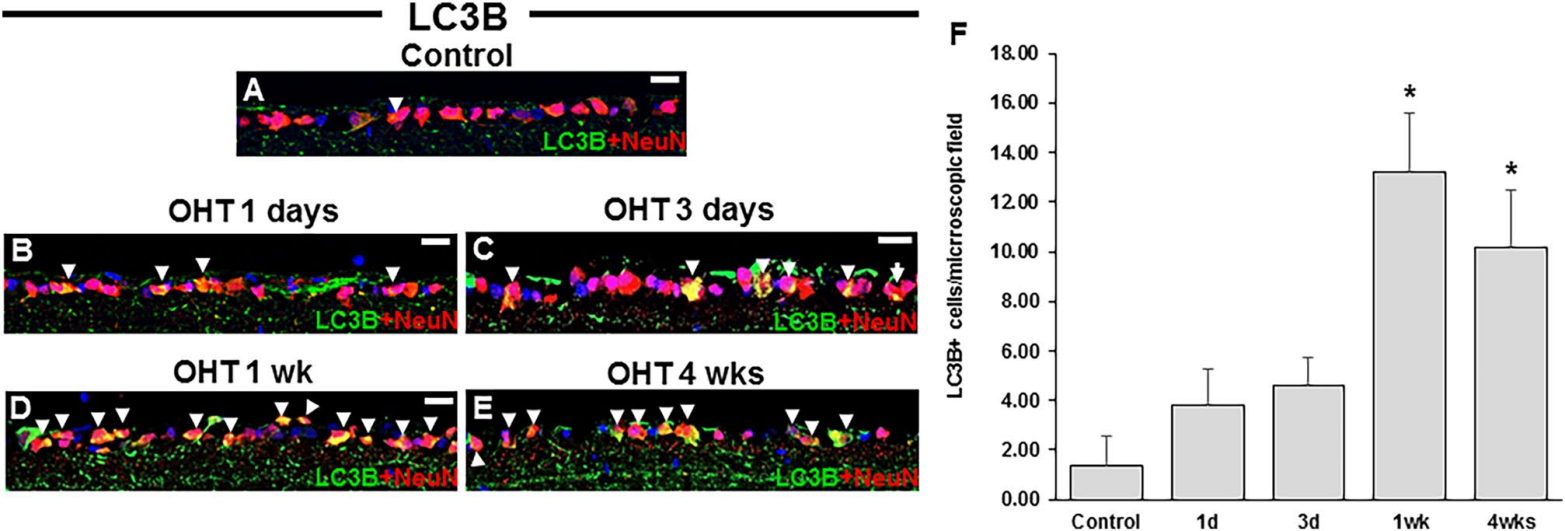
Time-course of LC3B expression in the eyes of the rats after circumlimbal suture placement. (**A**–**F**) In control eyes, scarce LC3B expression was colocalized with NeuN, an RGC marker (arrowhead) (**A**). One day after circumlimbal suture application, mild increased expression of LC3B in RGCs was observed (**B**), and further gradual increase in LC3B expression was detected in RGCs 1 week and 4 weeks after circumlimbal suturing (**C**–**E**), which was statistically significant at 4 weeks compared to non-OHT control (**F**). Data are expressed as mean ± SEM. *p < 0.05 versus control (non-OHT) retina; statistical significance was determined using the Student’s *t*-test. Scale bar, 20 µm.

Immunohistochemical staining for microtubule-associated protein light chain 3 (LC3) B, a well-known biochemical marker for autophagy initiation, was performed to assess autophagy induction in RGCs. In control eyes, scarce LC3B expression was visible in the cytoplasm of RGCs (Fig. 2A). Upon IOP elevation, the levels of LC3B in the cytoplasm of RGCs were also increased (Fig. 2B), followed by a further gradual increase up to 4 weeks after circumlimbal suturing (Fig. 2C–E). These findings suggest autophagy induction at later time points after IOP increase. Interestingly, there was also an increase in LC3B expression in the retinal nerve fiber layer of retinas from OHT eyes with similar time-dependent pattern shown in RGCs, which may be indicative of autophagy activation in RGC axons upon ocular hypertensive insult.

**Figure 3:**
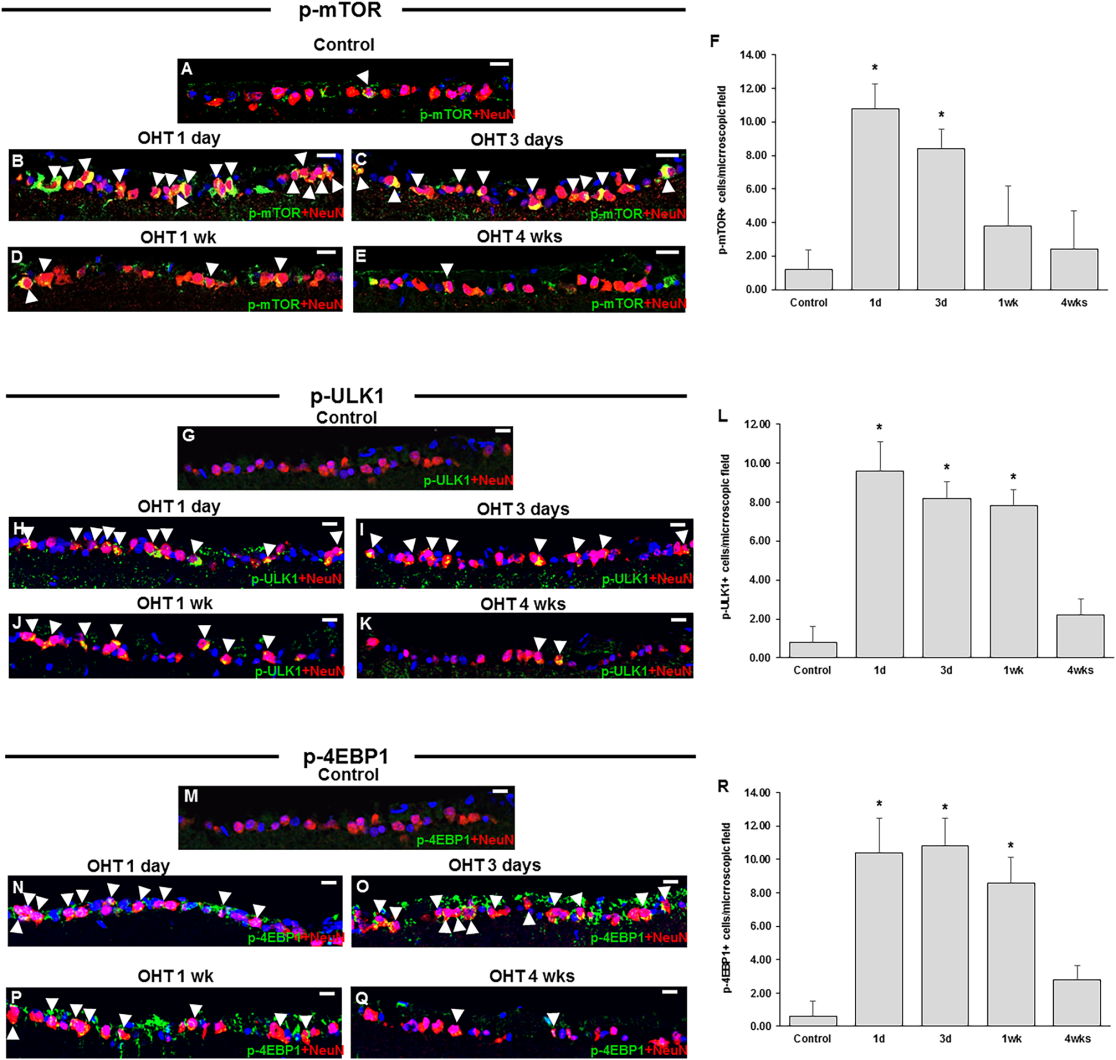
Time-dependent changes in expression of p-mTOR and its downstreatm targets, p-ULK1 and p-4EBP1 upon ocular hypertensive insult. (**A**–**F**) Immunostaining of the phosphorylated mammalian target of rapamycin (p-mTOR) showed limited colocalization with NeuN in control eyes (**A**), suggesting a low baseline mTOR activity. Upon OHT insult, significantly increased mTOR expression was detected in RGCs 1 day after circumlimbal suture application (**B**,**F**), which then gradually decreased until 4 weeks after the surgery (**C**–**F**). (**G**–**R**) Immunostaining of p-ULK1 (**G**–**L**) and p-4EBP1 (**M**–**R**) also showed similar time course pattern that was observed for p-mTOR, significant increase at day 1 of circumlimbal suturing and gradual decrease until 4 weeks of the experiment. Data are expressed as mean ± SEM. *p < 0.05 versus control (non-OHT) retina; statistical significance was determined using the Student’s *t*-test. Scale bar, 20 µm.

Autophagy induction was also assessed by immunohistochemical staining for the phosphorylated mammalian target of rapamycin (p-mTOR), the levels of which inversely correlate with autophagy activity. Low baseline p-mTOR levels were detected in the retinas of control animals (Fig. 3A). However, 1 day after circumlimbal suture application, p-mTOR levels were markedly increased (Fig. 3B), which then gradually decreased until 4 weeks after the surgery (Fig. 3C–F).

**Figure 4:**
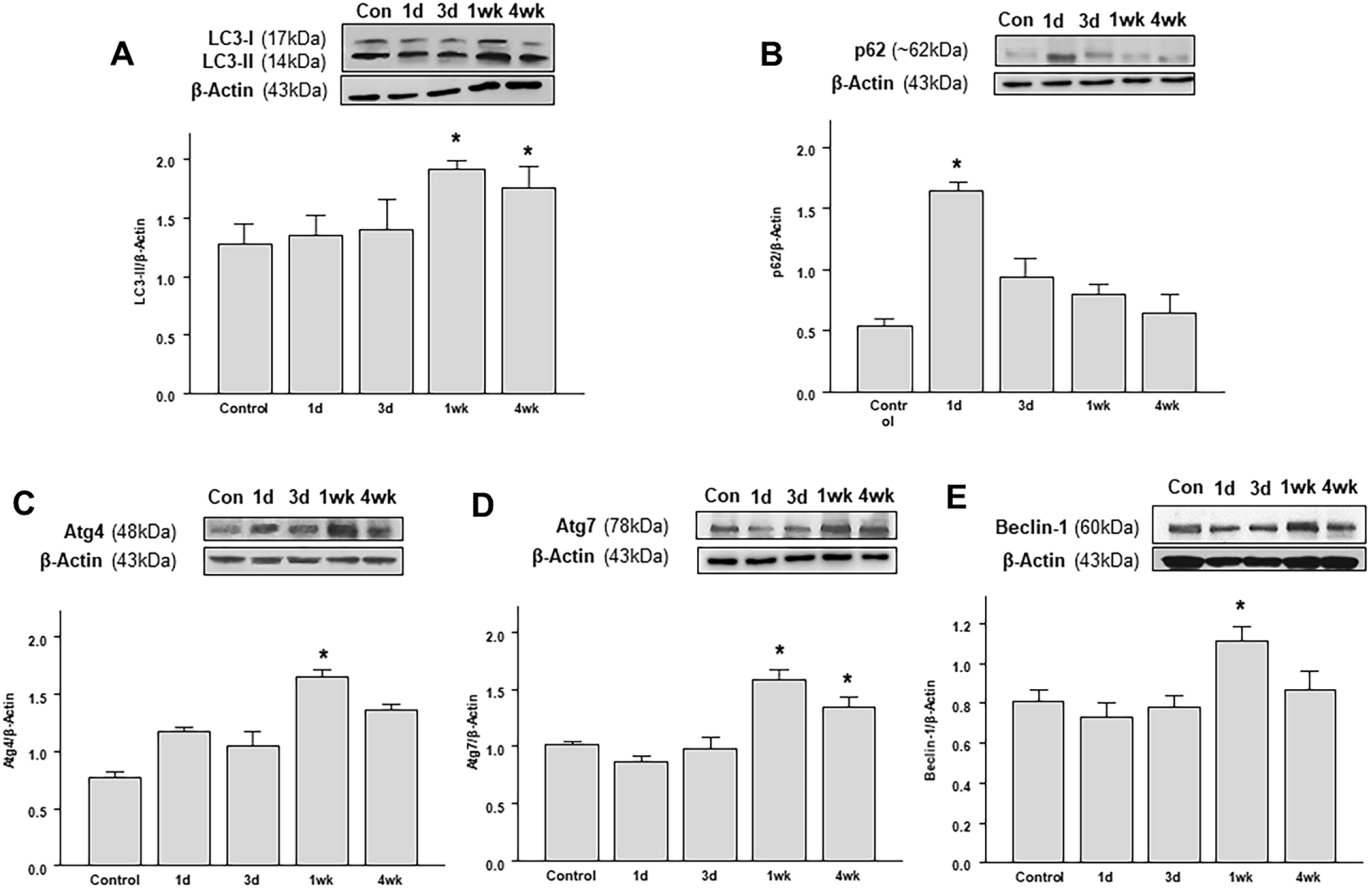
Time-course of autophagy-related protein levels in the retinas of the rats after circumlimbal suture application. (**A**) Western blotting showing a decrease in the LC3-II/LC3-I ratio in the retinas of the rats 1 day after the surgery detected compared to that in control animals. 1 and 4 weeks after the circumlimbal suture placement, the LC3-II/LC3-I ratio significantly increased compared to control rats, indicating a late autophagy induction after the OHT insult. (**B**) Immunoblotting for p62, another important autophagic marker, showed increased protein levels 1 day after circumlimbal suturing, which decreased to baseline levels 1 and 4 weeks later. (**C**,**D**) Western blotting for ATG4 (**C**) and ATG7 (**D**) revealed a significant increase in their protein levels 1 and 4 weeks after circumlimbal suture placement, while no significant changes were observed one and 3 days after the surgery compared to contralateral control eyes. (**E**) Time-course of Beclin-1 expression in OHT retinas showed similar to ATG4 and ATG7 pattern, with no significant changes in the protein levels one and 3 days after the surgery and a profound increase 1 week after the surgery. Histograms represent the densitometric values of the protein bands shown in Western blotting. Data are normalized to β-actin and expressed as mean ± SEM. *p < 0.05 versus control (non-OHT) retina; statistical significance was determined using the Student’s *t*-test (see supplementary material).

Activated mTOR and mTOR complex 1 (mTORC1) phosphorylate and inhibit Unc51-like kinase 1 (ULK1) and 4E-binding protein 1 (4EBP1), thereby suppressing autophagy induction. Therefore, we further assessed p-ULK1 and p-4EBP1 levels in RGC layer using immunohistochemical staining. Expression of p-ULK1 (Fig. 3G–L) and p-4EBP1 (Fig. 3M–R) in RGCs showed similar time course pattern observed for p-mTOR staining, suggesting activation of mTOR signaling pathway at early time points after circumlimbal suturing.

### Changes in the levels of autophagic markers in OHT eyes

To quantitatively investigate the activation of autophagy, Western blotting analyses for autophagy markers were conducted. During the formation of a mature autophagosome, the cytosolic protein LC3-I is converted into LC3-II, which is attached to the stable double-membrane of the autophagosome^22^. For quantification, it is highly recommended to calculate LC3-II/β-actin ratio to monitor autophagy activity^23^. We found that the IOP elevation resulted in a significant decrease in the LC3-II/ β-actin ratio 1 day after the surgery. Subsequently, the LC3-II/ β-actin ratio significantly increased 1 week after circumlimbal suturing and remained elevated for up to 4 weeks (Fig. 4A), indicating a late autophagy activation after IOP elevation. This time course of change was similar to that was observed in immunohistochemistry analysis for LC3B, but with mild discrepancy since Western blot analysis used the whole retinal tissue. Another important marker for autophagy is the ubiquitin-binding scaffold protein p62, which attaches substrates to LC3 for autophagic degradation. Therefore, increased autophagic activity results in a decrease in p62 levels. In our OHT animal model, significantly elevated p62 levels were observed 1 day after circumlimbal suturing, while its levels between 1 and 4 weeks after the surgery decreased to the baseline level (Fig. 4B). These findings suggest that autophagy flux was decreased early after OHT development.

**Figure 5:**
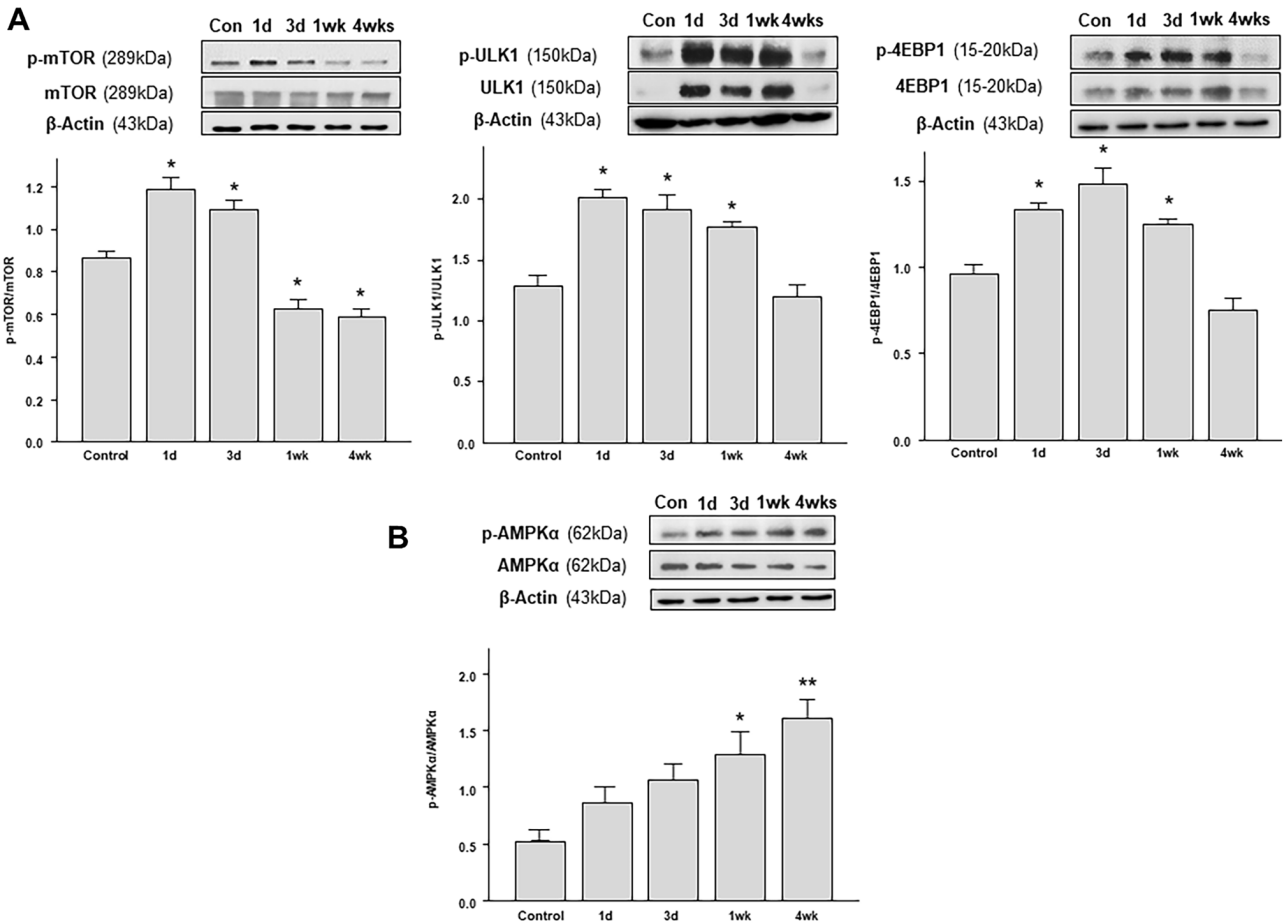
Time-dependent changes in mTOR signaling pathway and AMPK pathway activation in OHT retinas after circumlimbal suture placement. (**A**) Western blotting analyses showed a significant increase in the p-mTOR/mTOR ratio 1 day after the surgery, which then gradually decreased until 4 weeks after the surgery, suggesting activation of the mTOR pathway early after the OHT insult. The protein levels of the downstream targets of mTOR signaling pathway ULK1 and 4-EBP1 were investigated, revealing increased p-ULK1/ULK1 and p-4EBP1/4EBP1 ratios one and 3 days, as well as 1 week after the suture. Both ratios decreased 4 weeks after the surgery. (**B**) AMPK phosphorylation (shown as p-AMPK/AMPK ratio exhibited an opposite to mTOR pathway pattern, with a time-dependent increase until 4 weeks after the surgery. Histograms represent the densitometric values of the protein bands shown in Western blotting. Data are expressed as mean ± SEM. *p < 0.05 versus control non-OHT retina; statistical significance was determined using the Student’s *t*-test (see supplementary materials).

Furthermore, changes in ATG protein levels were analyzed by Western blotting. ATG4 mediates the processing and lipidation of LC3, while ATG7 promotes LC3 conjugation with phosphatidylethanolamine. The protein levels of both ATG4 (Fig. 5A) and ATG7 (Fig. 5B) were elevated 1 and 4 weeks after OHT development, while no significant changes in their levels were observed one and 3 days after the surgery. Beclin-1 (ATG6) is an essential player in the autophagosome formation process. Similar to other ATG proteins, a significant increase in Beclin-1 level was observed 1 week after the surgery (Fig. 5C).

### Changes in the activation of mTOR and AMPK pathways are associated with autophagic activity in OHT eyes

**Figure 6:**
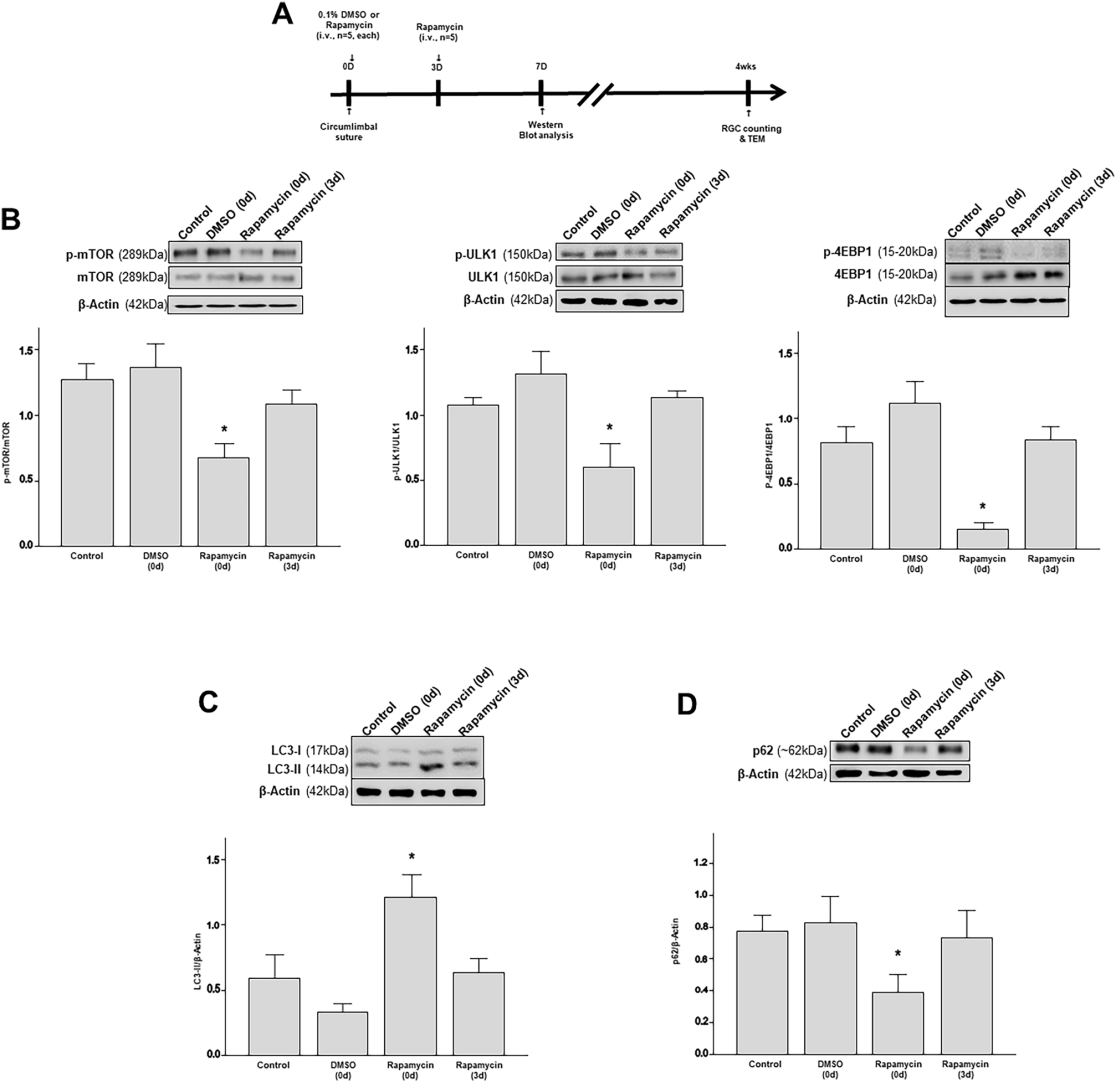
Time-course of mTOR signaling pathway and autophagy activation in the retina after rapamycin treatment. (**A**) Intravitreal rapamycin injection was performed immediately after circumlimbal suture application (day 0) and day 3, and mice were killed at 1 week for Western blot analysis or 4 weeks for evaluation of RGC survival and TEM imaging. (**B**) Intravitreal rapamycin injection at day 0 significantly decreased the levels of p-mTOR, p-ULK1, and p-4EBP1 in OHT retinas compared to control and DMSO injected retinas. However, intravitreal rapamycin injection 3 days after OHT insult did not significantly affect p-mTOR, p-ULK1, or p-4EBP1 level. (**C**,**D**) Intravitreal rapamycin injection (day 0) in OHT eyes significantly reduced the p62 (**C**) and LC3-II (**D**) levels compared to control and DMSO injected OHT eyes, suggesting autophagy induction early after rapamycin injection. Histograms represent the densitometric values of the protein bands shown in Western blotting. Data are normalized to β-actin (for LC3-II and p62) and expressed as mean ± SEM. *p < 0.05 versus control DMSO injected retina; statistical significance was determined using the Student’s *t*-test (see supplementary materials).

We found a significant increase in the p-mTOR/mTOR ratio 1 day after the application of circumlimbal suture, which subsequently gradually decreased for up to 4 weeks after the surgery (Fig. 6A). Similar time-dependent changes were observed in the downstream targets of mTOR signaling pathway p-ULK1 (Fig. 6B) and p-4EBP1 (Fig. 6C), the levels of which were elevated at day 1 and 3, as well as 1 week after the surgery, followed by a decrease 4 weeks after OHT development.

The activity of the autophagy-promoting AMP-activated kinase (AMPK), which is inversely correlated with mTOR activity, was also investigated by Western blotting analyses. AMPK phosphorylation exhibited a time-dependent increase until 4 weeks after the surgery (Fig. 6D), showing an opposite to mTOR pathway targets pattern.

### Effects of intravitreal rapamycin injection on RGC survival

**Figure 7:**
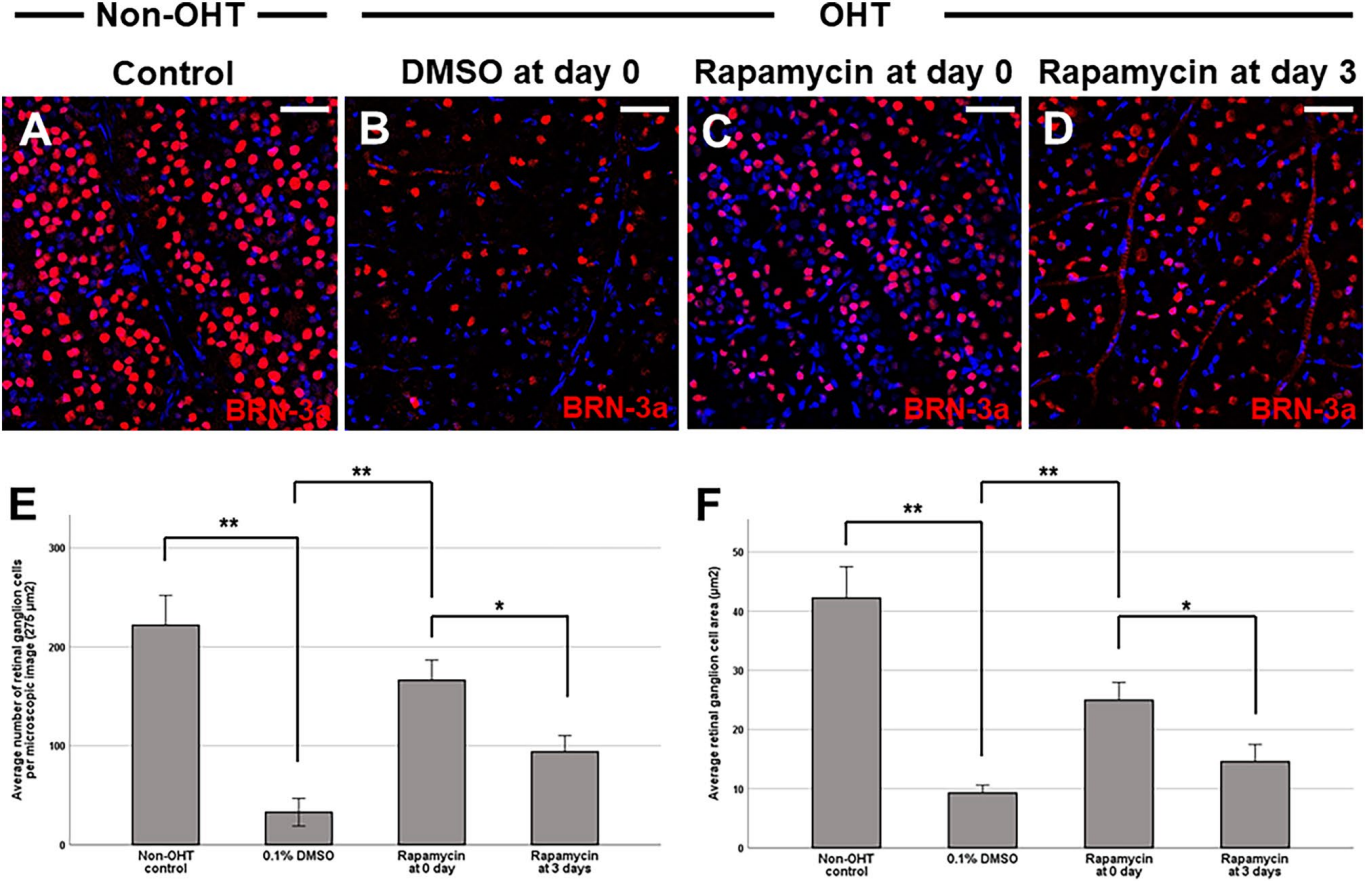
Early Intravitreal rapamycin injection promotes RGC survival in the circumlimbal suture OHT rat model. (**A**,**B**,**E**) Retinal whole-mount immunostaining for BRN-3a showed significantly decreased number of RGCs in OHT eyes with intravitreal 0.1% DMSO injection after 4 weeks of circumlimbal suture compared to the non-OHT control group. (**B**–**F**) Intravitreal rapamycin injected 0 and 3 days after circumlimbal suture application significantly increased the RGC numbers and cell sizes compared to the control group (intravitreal 0.1% DMSO injection). (**C**–**F**) The number and cell size of RGCs between OHT eyes with intravitreal rapamycin injection at 0 and 3 days after circumlimbal suture placement also showed significant difference, intravitreal rapamycin injected 0 day showing more enhanced RGC survival effect. Representative images were taken from middle (2 mm from the optic disc) regions of the retinal whole mounts. Data are expressed as mean ± SEM. **p < 0.01, *p < 0.05. Scale bar: 40 µm.

To assess the effects of autophagy induction in the retina at different time-points following the circumlimbal suture application, intravitreal rapamycin injection was performed immediately after the suture placement (day 0) and 3 days after the surgery, while intravitreal 0.1% DMSO injection was performed at day 0 for vehicle control group (Fig. 7A). Since autophagy activity seemed to be upregulated from 1 week after the surgery in western blot analysis, we performed intravitreal rapamycin injection only at day 0 and 3 after suture placement. To confirm that there was no significant downstream effect of IOP after intravitreal injection, IOP was monitored at 1, 6, 12, and 24 h after intravitreal injection. There was no statistically significant decrease in IOP after intravitreal injection compared to that measured before the injection (IOP at 1 h after intravitreal injection at day 0 and day 3 : 51 ± 7 mmHg and 24 ± 3 mmHg, respectively) (Supplementary figure S1).

To confirm mTOR pathway inhibition upon intravitreal rapamycin injection, p-mTOR/mTOR, p-ULK1/ULK1, and p-4EBP1/4EBP1 ratios were investigated by Western blotting. As shown in Fig. 7, intravitreal rapamycin injection at day 0 showed the most potent inhibition of mTOR phosphorylation, as well as ULK1 and 4EBP1 (Fig. 7B). However, intravitreal rapamycin injection 3 days after circumlimbal suture application led to subtle decrease in p-mTOR/mTOR ratio level, which was not statistically significant compared to the control. Intravitreal rapamycin injection immediately after the suture placement significantly reduced and increased the p62 protein and LC3-II/β-actin level, respectively (Fig. 7C), further supporting mTOR inhibition and subsequent autophagy induction early during OHT development.

**Figure 8:**
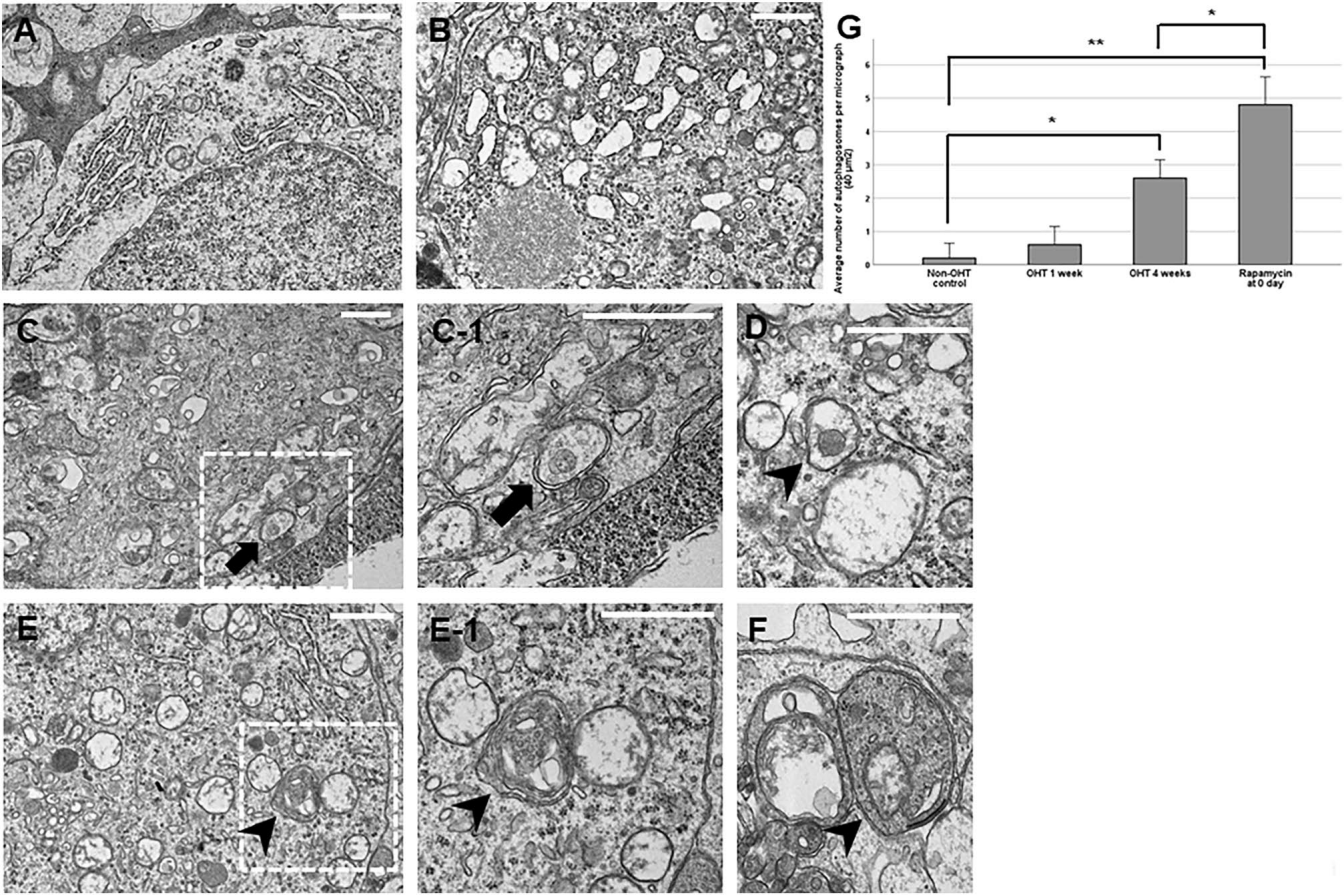
Ultrastructural analyses of retinas after OHT insult. (**A**,**B**) Transmission electron microscopy (TEM) revealed scarce number of autophagosome in the cytoplasm of RGCs from non-OHT control (**A**) and OHT 1 week retinas (**B**), with profound dilation of endoplasmic reticulum in OHT 1 week RGCs (**B**). (**C**,**D**) The cytoplasm of RGCs from rats subjected to intravitreal DMSO injection contained a several number of immature (**C**) and mature autophagosomes (**D**), characterized by a double limiting membrane. (**E**,**F**) The cytoplasm of RGCs from rats subjected to intravitreal rapamycin injection (day 0) contained several closed, mature autophagosomes (arrowheads), indicative of autophagy activation. The number of autophagosomes in RGCs of non-OHT control was 0.22/40 μm^2^, which significantly increased to 2.63/40 μm^2^ at 4 weeks of OHT induction (**G**). Intravitreal rapamycin injection at day 0 resulted in significant increase in the number of autophagosomes (4.89/40 μm^2^) in the cytoplasms of RGCs compared to both non-OHT and OHT 4 weeks eyes. Boxed area in (**A**) and (**B**) is shown in high magnification in A-1 and B-1, respectively. Data are expressed as mean ± SEM. **p < 0.01, *p < 0.05 vs. non-OHT control. Scale bar: 1000 nm.

We also investigated the effects of intravitreal rapamycin injection at different time-points on RGC survival. We found that intravitreal rapamycin injection at days 0 and 3 after circumlimbal suture application resulted in a significant increase in RGC numbers in OHT eyes compared to the control group (intravitreal DMSO injection; Fig. 8A–D). Also, intravitreal rapamycin injected at 0 day after suture placement showed significantly enhanced RGC survival effect compared to when it was given at 3 days after the surgery (Fig. 8E), not only in number but also in cell size. Considering that mTOR inhibition triggers autophagy induction, autophagy induction early during OHT development might have potent neuroprotective effects by promoting RGC survival.

### Ultrastructural changes after circumlimbal suture application and intravitreal rapamycin injection

Autophagy induction was further confirmed by the investigation of the presence of autophagosome by transmission electron microscopy, which can be identified as double-membrane vesicles containing cytosolic substrates^5^. Scarce number of autophagosome, 0 to 1, was found in the cytoplasm of RGCs from non-OHT control and OHT 1 week retinas (Fig. 8A,B), with profound dilation of endoplasmic reticulum in OHT 1 week RGCs. The cytoplasm of RGCs from rats 4 weeks after circumlimbal suture placement contained a several number of mature and immature autophagosomes (Fig. 8C,D), suggesting early phase autophagy or impaired autophagic flux. On the other hand, the cytoplasm of RGCs from OHT rats subjected to intravitreal rapamycin injection (day 0) contained numerous mature autophagosomes containing amorphous electron-dense material (Fig. 8E,F), suggesting autophagy activation. We also counted autophagosomes in 50 micrographs (2000 μm^2^) in total to quantify the number of autophagosomes in the cytoplasm of RGCs for each group. The number of autophagosomes in RGCs of non-OHT eyes was 0.22/40 μm^2^, which significantly increased to 2.63/40 μm^2^ at 4 weeks of OHT induction (Fig. 8G). Intravitreal rapamycin injection at day 0 resulted in significant increase in the number of autophagosomes (4.89/40 μm^2^) in the cytoplasms of RGCs compared to both non-OHT and OHT 4 weeks eyes.

## Discussion

We demonstrated that autophagic activity differed at different time points during OHT development. Using a circumlimbal suture OHT rat model, we found that the autophagic flux was enhanced 1 and 4 weeks after OHT induction, suggesting that autophagy was a late event during OHT development. These findings were confirmed by immunohistochemistry, Western blotting, and TEM analyses. Furthermore, autophagy induction at different time points after circumlimbal suture placement using intravitreal rapamycin injection showed that autophagy induction immediately after surgery was more potent in promoting RGC survival compared to when rapamycin was injected at later time points. This finding suggests that autophagy induction early during OHT development might have potent neuroprotective effects on RGC survival in OHT.

While autophagy is an essential process for maintaining cellular homeostasis and adapting to various stressors, its effects in neurodegenerative conditions can be either protective or detrimental, depending on the circumstances. Neuronal autophagy induction has been reported in various stress environments^24–26^. Previous studies have shown that blocking autophagy might lead to neurodegenerative defects in neurons and axons^11,27^. Moreover, several studies have suggested that impairments in autophagy might contribute to the development of Alzheimer’s disease^28–30^, Parkinson’s disease^31,32^, and amyotrophic lateral sclerosis^33,34^. Despite evidence regarding the potential therapeutic effects of autophagy induction in neurodegenerative diseases, autophagy has also been reported to enhance neurodegeneration in certain circumstances^35–37^. Hence, the neuroprotective effects of autophagy in neuronal degeneration have remained a matter of debate.

Glaucoma is a neurodegenerative disease that occurs in the optic nerve and is characterized by loss of RGCs and their axons, often accompanied by IOP elevation. Similar to neurodegenerative diseases affecting the brain, activation of autophagy in RGCs has been demonstrated in various glaucoma animal models, which suggests the presence of time-dependent patterns in autophagic activity. Using an episcleral vein electrocauterization model, Park et al. demonstrated that the levels of the autophagy markers LC3-II and Beclin-1 were increased in RGCs between 1 and 2 weeks after IOP elevation, and remained high until after 8 weeks^19^. Another study using a chronic OHT rhesus monkey model also showed increased levels of LC3-II, Beclin-1, and autophagosomes in RGCs^38^. On the other hand, studies that have used retinal ischemia/reperfusion models, which are characterized by an acute elevation of IOP by pressurizing the anterior chamber, have reported a decrease in LC3-II and Beclin-1 level, as early as at 1 h after the insult, which then returned to normal levels after 6 to 24 h^39,40^. An early decrease in autophagic activity has been suggested to contribute to RGC death in ischemia/reperfusion models. In this study, we performed circumlimbal suturing to increase IOP in rats with OHT^20,41,42^, which is achieved by suppression of aqueous outflow mediated by ocular compression. The protein levels of autophagy markers, including LC3-II, Beclin-1, and ATG proteins (ATG4 and ATG7), were elevated late, usually between 1 and 4 weeks, while decreased or similar to control levels of autophagy markers were observed 1 and 3 days after the OHT induction. Furthermore, the transient increase in p62 and decrease in ATG protein levels 1 day after the circumlimbal suture application indicated impairments in autophagy early during OHT development, suggesting that early impairment in autophagy might contribute to RGC death during OHT. However, while the pattern of IOP elevation in this model (Fig. 1) is reminiscent of that in acute rather than chronic ocular hypertensive glaucoma, further studies using more typical chronic OHT animal models are necessary to exactly evaluate the time course of autophagy activity in the retina under chronic OHT condition.

Autophagy is negatively regulated by the mTOR pathway, through the phosphorylation and subsequent inhibition of the ULK1 complex^43,44^. In this study, we found decreased levels of autophagy markers 1 day after OHT induction concurred with increased phosphorylation levels of mTOR and its two main downstream targets ULK1 and 4EBP1. Moreover, the increase in autophagy activity 4 weeks after surgery was accompanied by a gradual decrease in the mTOR pathway activity. This time-dependent pattern in mTOR activity inversely coincided with that of AMPK activation, which inhibits the mTOR pathway^44^. These findings suggest the critical role of the mTOR pathway in autophagy regulation in our circumlimbal suture OHT model, and that autophagy could be modulated by interfering with the mTOR signaling pathway.

Several studies have shown that the administration rapamycin, an autophagy inducer, may have neuroprotective effects on RGC survival in glaucoma. Using a chronic OHT model, Su et al. reported that intraperitoneal rapamycin injection suppressed RGC apoptosis^16^. Consistently, Russo et al.^40^ showed that activating autophagy using rapamycin or fasting promoted RGC survival in a retinal ischemia/reperfusion model. On the other hand, some studies have shown that inhibiting autophagy with 3-MA has neuroprotective effects on RGC survival in both chronic OHT models^19^ and ischemia/reperfusion models^17^, complicating the role of autophagy in RGC survival. Due to these discordant results, we hypothesized that the role of autophagy in RGC survival might differ according to the stage during OHT development. Thus, we investigated the neuroprotective effects of rapamycin injected intravitreally at different time points after circumlimbal suturing. Our results showed that intravitreal administration of rapamycin immediately after the suture suppressed the mTOR pathway and induced autophagy, as indicated by the decrease in p-mTOR and p62 levels. Early autophagy induction also enhanced RGC survival in the retinas of OHT rats. Therefore, at least in our circumlimbal suture OHT model, early induction of autophagy enhanced RGC survival upon OHT induction, supporting the neuroprotective effects of autophagy in RGCs. However, as rapamycin may affect other intracellular pathways to produce pro-survival factors^45,46^, future studies using more specific autophagy-modulating agents are needed to confirm the neuroprotective effects of rapamycin shown in this study.

One concern is that in this study, we only performed single intravitreal to observe its RGC survival effects. In a previous study, it has been reported that one-time intravitreal rapamycin injection resulted in sustained release of rapamycin into vitreous humor of rabbit eyes until 2 months after injection^47^. Although in this study we used different dose of rapamycin, we may expect that intravitreally injected rapamycin may have resulted in sustained release into the vitreous humor of rat eyes. However, there should be further studies regarding dose-dependent effect of intravitreally administered rapamycin in OHT eyes, as well as their vitreous concentration at the time of analysis.

In summary, autophagy was induced in the retinas of OHT eyes between 1 and 4 weeks after OHT induction, while autophagy activity was impaired early after OHT induction. Moreover, intravitreally administration of rapamycin immediately after the application of the circumlimbal suture exhibited the most potent neuroprotective effect on RGCs, indicating that autophagy induction early during OHT development may have pro-survival effects in RGCs. Thus, the autophagy machinery might serve as an important therapeutic target to promote neuroprotection in the management of glaucoma.

## Materials and methods

### Animals

Sixty-six male Sprague–Dawley rats, aged 7–8 weeks (Orient Bio, Sungnam, Republic of Korea), were used in this study. Animals were housed under standard conditions and a 12/12-h light/dark cycle with food and water ad libitum. Animal care and experimental procedures were carried out in compliance with the ARVO Statement for the Use of Animals in Ophthalmic and Vision Research, and this study was approved by the Animal Care Committee of Soonchunhyang University Bucheon Hospital (Permit Number: SCHBCA201708). Deep sedation in rats was performed by intraperitoneal injection of a mixture containing 40 mg/kg zolazepam/tiletamine (Zoletil; Virbac, Carros Cedex, France) and 5 mg/kg xylazine (Rompun; Bayer Healthcare, Leverkusen, Germany) before proceeding to any surgical procedures.

### Circumlimbal suture rat model of chronic ocular hypertension (OHT)

Baseline IOP readings were measured in animals with a rebound tonometer (TonoLab; iCare, Helsinki, Finland), and IOP was measured between 11 AM and 12 PM to exclude the effects of diurnal IOP variation. After baseline IOP measurements, animals were divided into OHT and non-OHT control groups. For animals in the OHT group, the circumlimbal suture technique was used to induce IOP elevation in one eye, as described previously^20,21^. Briefly, circumferential suturing (8/0 nylon) was performed around the globe at an approximate distance of 1.0–1.5 mm behind the limbus. The contralateral eye was left untreated. IOP was measured using TonoLab immediately, as well as 1, 2, 3, 7, 14, 21, and 28 days after suturing. The IOP was measured in awake animals without topical anesthesia, except for the measurement immediately after circumlimbal suture knotting, which was performed during recovery from sedation.

Twenty-four rats from OHT and six non-OHT group were used for cross-sectional immunohistochemistry analysis (OHT: n = 12, non-OHT: n = 3) and Western blot analysis (OHT: n = 12, non-OHT: n = 3). Eighteen rats from OHT group underwent intravitreal DMSO at day 0 (n = 6) or rapamycin injection at day 0 (n = 6) and day 3 (n = 6), and used for whole mount immunohistochemistry (n = 3 from each group) or Western blot analysis (n = 3 from each group). Six rats from non-OHT control group was used for whole mount immunohistochemistry (n = 3) and Western blot analysis (n = 3) to compare the effect of intravitreal rapamycin injection.

### Intravitreal administration of rapamycin

Intravitreal rapamycin (37094; Sigma-Aldrich) injection was performed in eyes with OHT under anesthesia immediately after circumlimbal suturing (n = 6), as well as 3 days after the surgery (n = 6). The rats were anesthetized and eyes were visualized using a surgical microscope in a lateral position. After topical anesthesia with 0.5% proparacaine hydrochloride (Paracaine Eye Drops; Hanmi Pharm., Seoul, Republic of Korea) and pupil dilation with a mixture of 0.5% tropicamide and 0.5% phenylephrine hydrochloride (HCL) (Tropherine Eye Drops; Hanmi Pharm.), a sclerotomy was created using a 30-gauge needle at approximately 0.5–1.0 mm posterior to the limbus. Next, 2 µL rapamycin (50 ng/µL) dissolved in DMSO or 0.1% DMSO as vehicle control was injected intravitreally using a NanoFil syringe with a blunt 35 G needle (World Precision Instruments, Sarasota, FL) through the sclerotomy site.

### Tissue preparation

Ocular tissues were processed for further analyses, as described previously^48,49^. Briefly, rats were deeply anesthetized and intracardially perfused with 0.1 M phosphate-buffered saline (PBS) containing 150 U/mL heparin, followed by perfusion with 4% paraformaldehyde (PFA) in 0.1 M PBS. Subsequently, the eyes were enucleated, and a 360° sclerotomy around the limbus was done to obtain the posterior segment of eyeball. Tissues were fixed in 4% PFA, followed by overnight incubation in 30% sucrose (in PBS) and embedding in Optimal Cutting Temperature compound. Sections were cut serially with 10 µm thickness and mounted on adhesive microscope slides (Histobond; Marienfeld-Superior, Lauda-Königshofen, Germany). For preparing retinal whole mounts, posterior eyecups were fixed in 4% PFA and flattened by making four equidistant cuts.

### Immunohistochemistry (IHC)

Sections of retinas were permeabilized in 0.1% Triton X-100 containing 5% goat serum for 1 h, followed by overnight incubation at 4 °C with the primary antibodies. Sections were then washed and incubated with for 1 h at room temperature with secondary antibodies: Alexa Fluor 488-conjugated donkey anti-rabbit IgG (Invitrogen, Carlsbad, CA) and Alexa Fluor 568-conjugated donkey anti-mouse IgG (Invitrogen). Nuclei were counterstained with 4,’6-diamidino-2-phenylindole dihydrochloride (DAPI, 0.1 mg/mL; Sigma-Aldrich, St. Louis, MO) for 3 min. Retinal whole mounts were also stained following the same procedure. Confocal microscopy (LSM510 Meta; Carl Zeiss, Oberkochen, Germany) was used to examine and photograph the samples, and images were processed using the ZEN Digital Imaging for Light Microscopy software suite (Carl Zeiss). For counting RGCs, images from the peripheral (3 mm from the optic disc, 12 images), middle (2 mm from the optic disc, 8 images) and central (1 mm from the optic disc, 4 images) regions of the whole mounts were used. The primary antibodies used for IHC are listed in Table 1.

**Table 1:** Sources and dilutions of primary antibodies.

Target	Supplier and catalog no	Method and dilution
LC3b	Novus Biologicals, NB100-2220	IF 1/200, WB 1/1000
BRN-3α	Chemicon, MAB1585	IF 1/100
Neu-N	Chemicon, MAB377	IF 1/1000
p-mTOR	Abcam, ab109268	IF 1/200, WB 1/1000
ATG7	Abcam, ab223365	WB 1/1000
Beclin-1	Cell signaling, 3495	WB 1/1000
4EBP1	Cell signaling, 9644	WB 1/1000
p-4EBP1(Thr37/46)	Cell signaling, 2855	WB 1/1000
ULK1	Cell signaling, 8054	WB 1/1000
p-ULK1(Ser757)	Cell signaling, 6888	WB 1/1000
AMPK	Cell signaling, 2603	WB 1/1000
p-AMPK(Thr172)	Cell signaling, 2535	WB 1/1000
ATG4B	Cell signaling, 5299	WB 1/1000
p62	Sigma, P0067	WB 1/1000

WB western blotting, IF immunofluorescence.

### Western blotting analyses

Extracted retinas were lysed in 80 µL ice-cold lysis buffer. Subsequently, the lysates were centrifuged for 15 min, 10,000×*g* and 4 °C, and supernatants were collected and assayed for protein concentration using a BCA protein assay kit (Thermo Scientific, Rockford, IL). An equal amount of total protein (20 μg) was seperated by SDS polyacrylamide gel electrophoresis and transferred onto nitrocellulose membranes. Using 5% skim milk, membranes were blocked for 1 h at room temperature, and overnight incubation was performed with the primary antibodies listed in Table 1. Then they were incubated with species-specific horseradish peroxidase (HRP)-conjugated goat IgG secondary antibodies (1:5000; Santa Cruz Biotechnology, Santa Cruz, CA) for 1 h at room temperature. Band signals were detected using an enhanced chemiluminescence system (ECL; Bio-Rad Laboratories, Hercules, CA), and autoradiographic films were scanned and digitized at 600 dpi. Band intensities were quantified using ImageJ software (National Institutes of Health, Bethesda, MD, USA). Western blots shown are representative of at least three independent experiments.

### Transmission electron microscopy (TEM)

Next, 4 weeks after circumlimbal suturing, rats were sacrificed and their eyes were enucleated. Neural retinas were separated from the posterior eyecups and fixed in Karnowsky fixative (2% glutaraldehyde, 2% paraformaldehyde, and 0.5% calcium chloride in 0.1 M phosphate buffer, pH 7.4) for 12 h at 4 °C. After fixation, neural retinas were washed with 0.1 M phosphate buffer for 2 h. Subsequently, tissues were fixed with 1% osmium tetroxide in 0.1 M phosphate buffer for 1 h at room temperature and dehydrated with ethanol. Samples were embedded in an Epon 812-propylene oxide mixture and sectioned (80 nm thick) using a Reichert Ultracut S ultratome (Leica, Nussolch, Germany). Sections were stained with 6% uranyl acetate and lead citrate and examined using a JEM-1011 transmission electron microscope (Jeol, Tokyo, Japan). TEM images were photographed using a Morada digital camera. For TEM image analysis, total of twelve rats were used from non-OHT (n = 3), OHT at 1 week (n = 3), OHT at 4 weeks (n = 3), and intravitreal rapamycin injected group (n = 3).

### Image analyses and statistical analyses

Data are shown as mean ± standard error, and statistical significance was determined using the Student’s *t*-test. Twelve cross-sections of the retina were used to quantify LC3B, p-mTOR, p-ULK1, and p-4EBP1 positive cells. Quantitative comparison of the number and cell size of RGCs among the treatment groups was performed using the ImageJ software, and the Kruskal–Wallis test with post hoc analyses were used to determine statistically significant differences among the groups. Statistical analyses were conducted using SPSS for Windows software (ver. 20.0; SPSS Inc., Chicago, IL). Differences at p < 0.05 were considered statistically significant.

## Supplementary Information

Supplementary Information.
