# Supplementary material for: Characterization of the role of autophagy in retinal ganglion cell survival over time using a rat model of chronic ocular hypertension
Source: Sci Rep. 2021 Mar 11;11:5767. doi: 10.1038/s41598-021-85181-x (PMC7952572; doi:10.1038/s41598-021-85181-x)
Supplement: Supplementary file 1 — Supplementary Information. [file 41598_2021_85181_MOESM1_ESM.docx]

**Characterization of the role of autophagy in retinal ganglion cell survival over time using a rat model of chronic ocular hypertension**

Si Hyung Lee, Kyung Sun Shim, Chan Yun Kim, and Tae Kwann Park.

**Supplementary Figures S1**

**
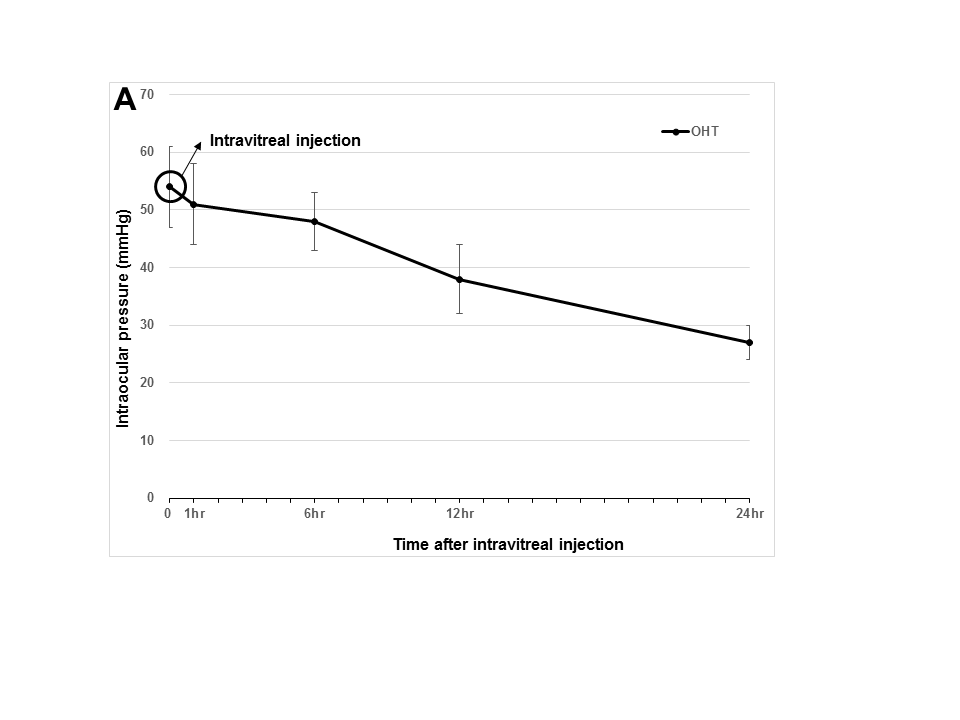
**

**
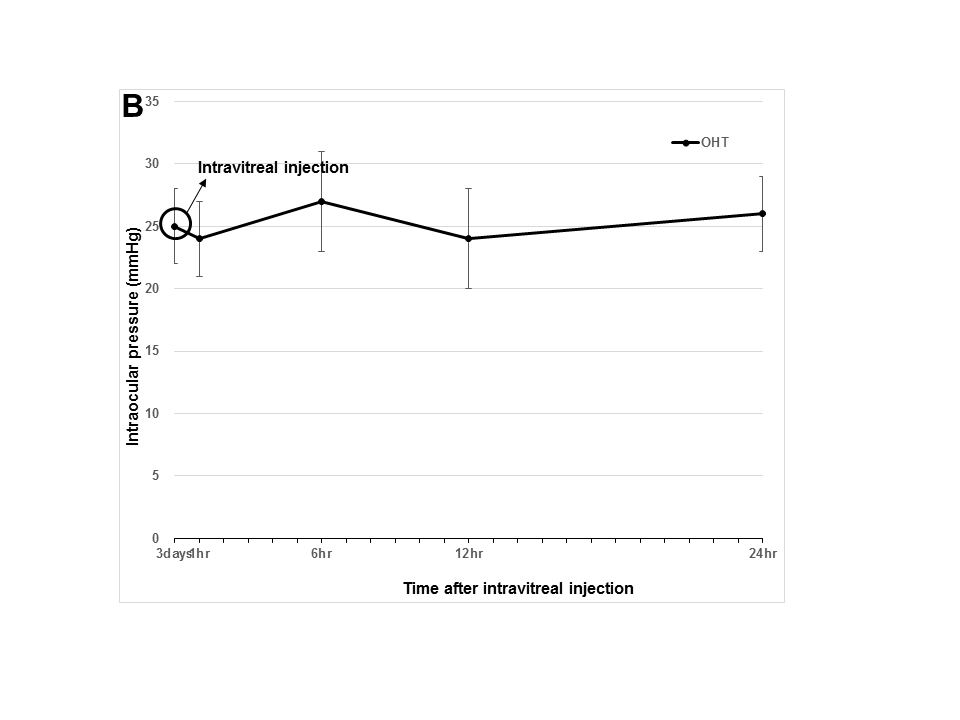
**

Time course of intraocular pressure (IOP) after intravitreal injection at day 0 (A) and at day 3 (B). No significant immediate downstream effect of IOP was noticed after intravitreal injection.

**Supplementary Figures S2**


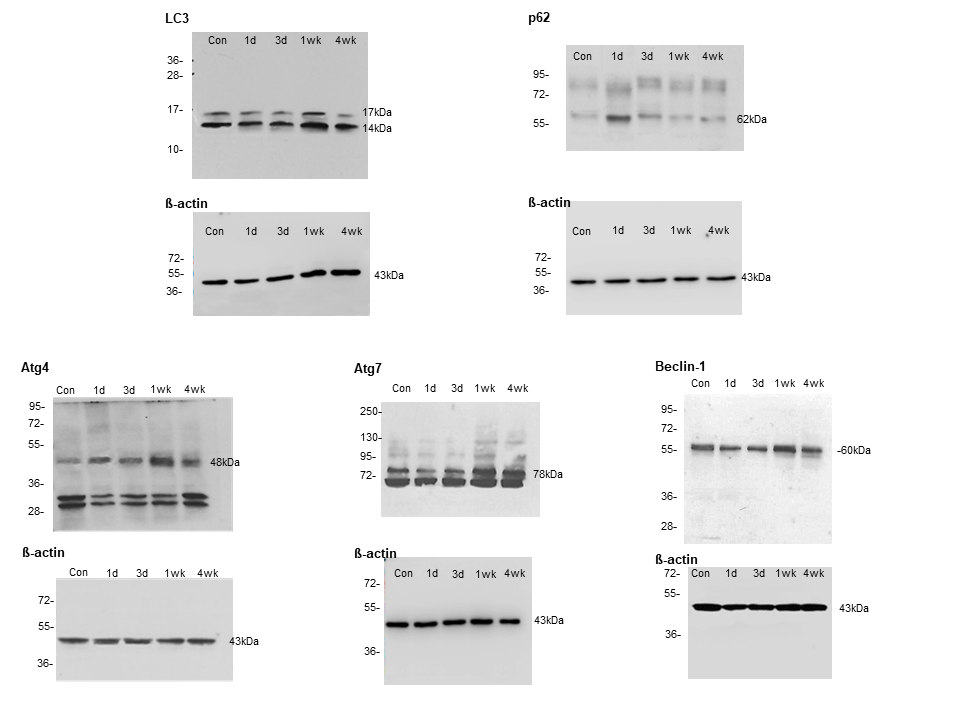


Full length blots of Figure 4.

**Supplementary Figures S3**


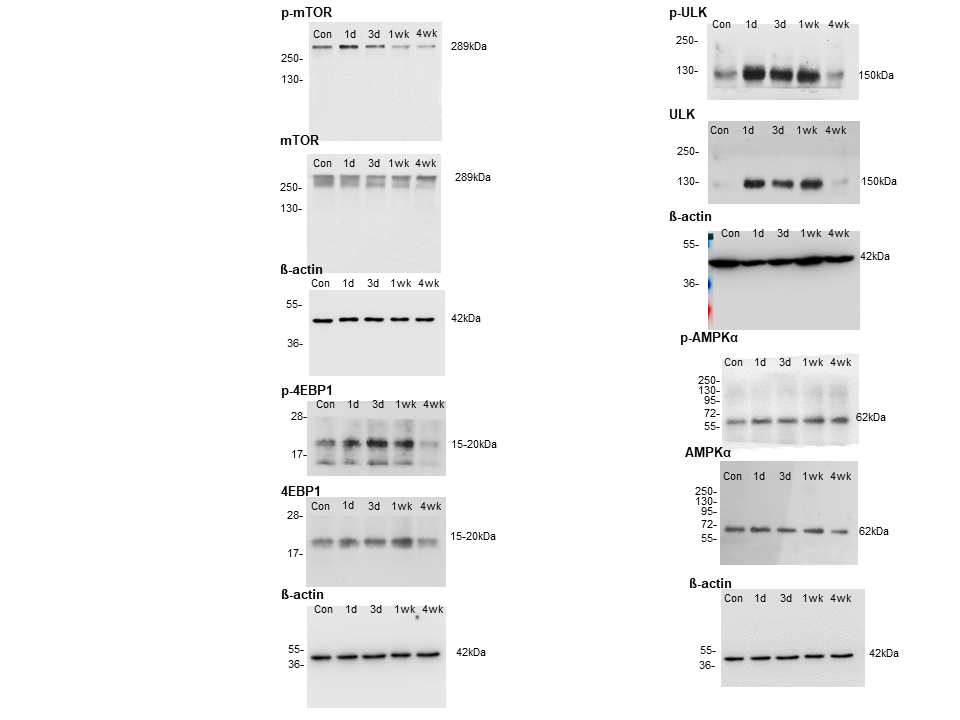


Full length blots of Figure 5.

. **Supplementary Figures S4**


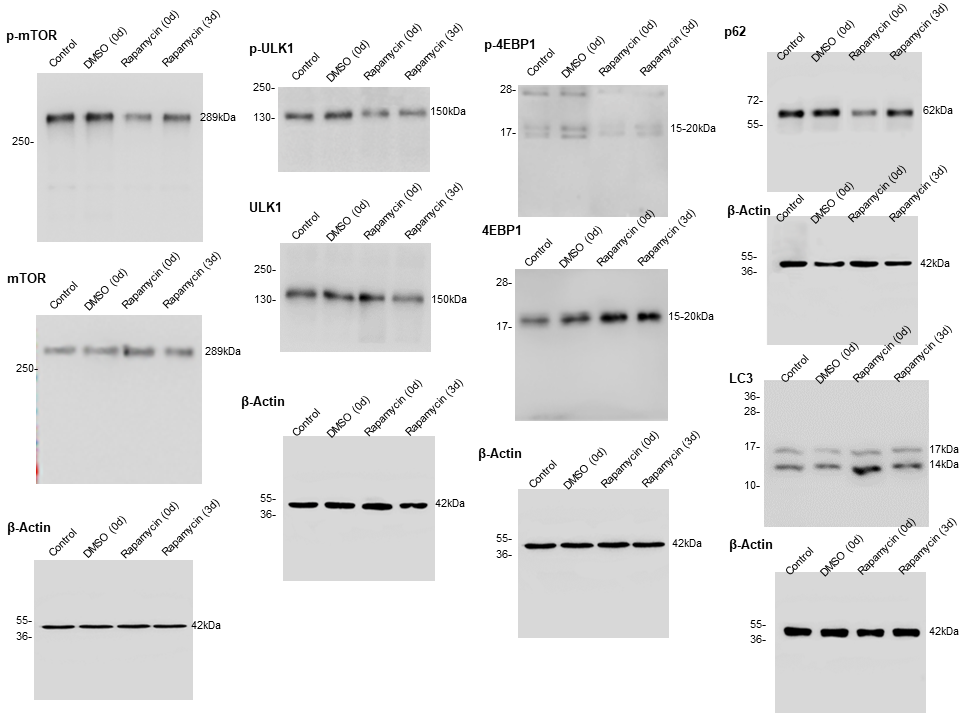


Full length blots of Figure 6.

. **Supplementary Figures S5**


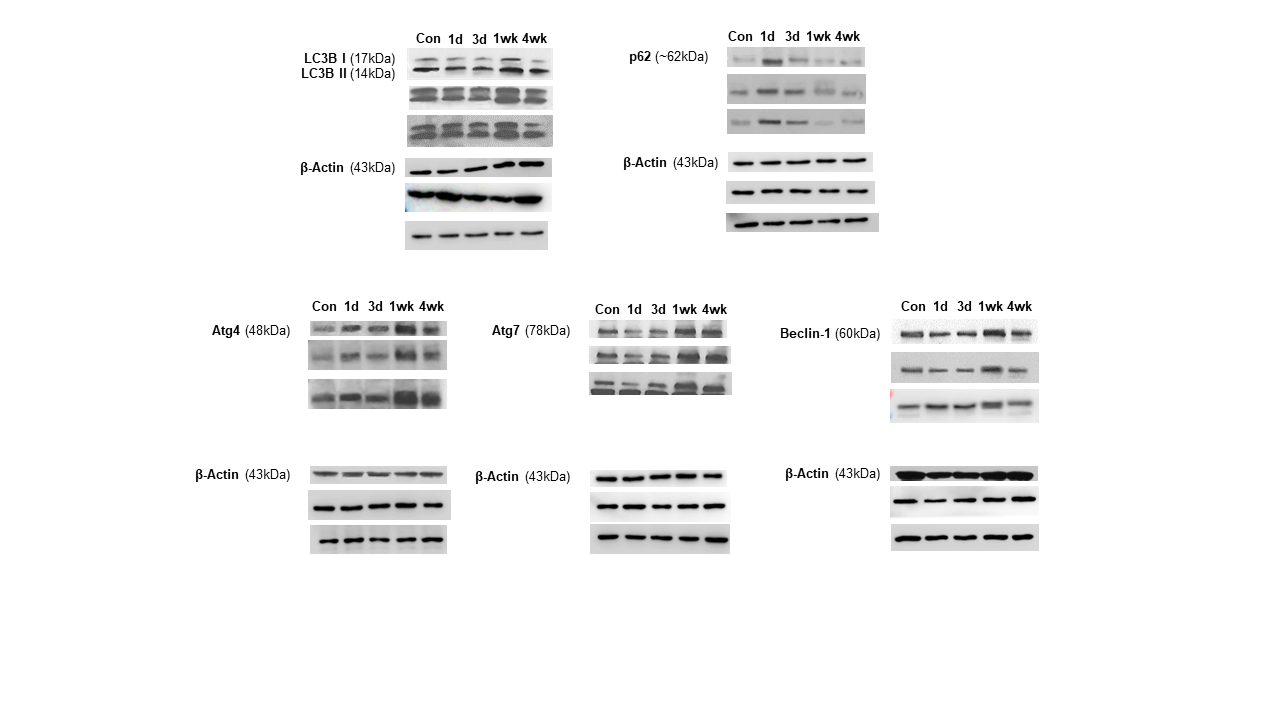


Western blot bands used for analysis for Figure 4.

**Supplementary Figures S6**


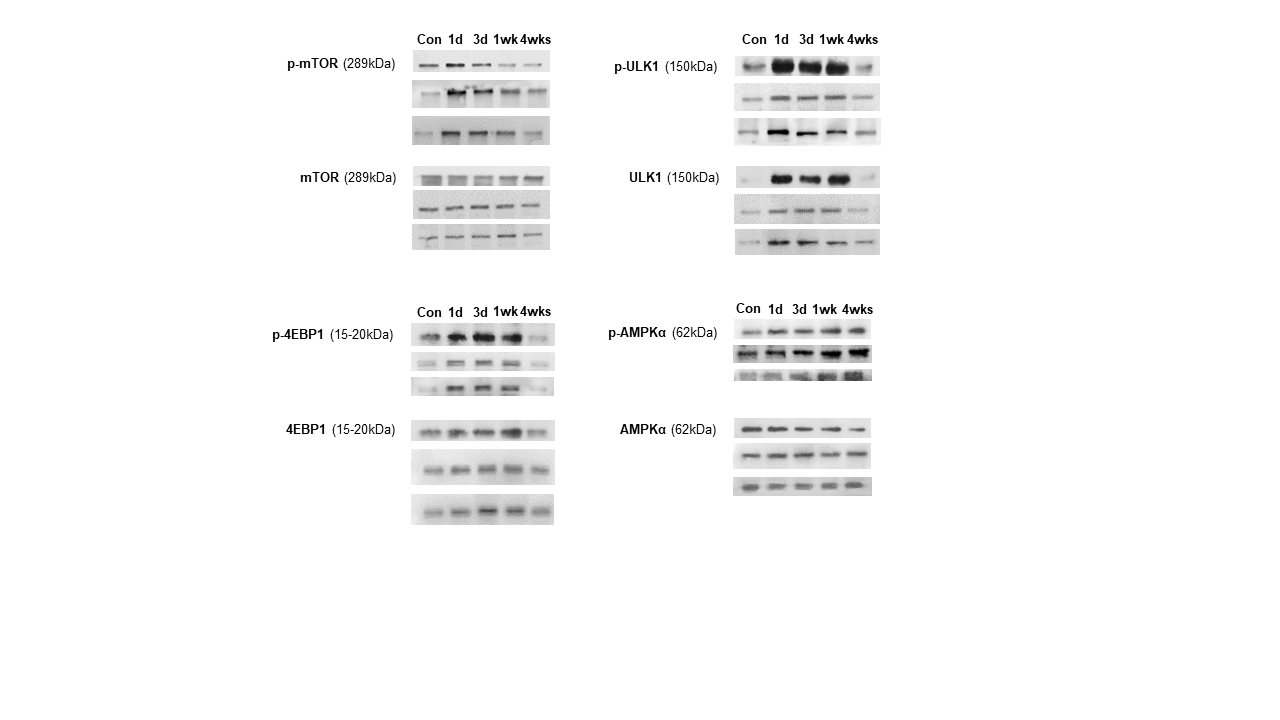


Western blot bands used for analysis for Figure 5.

**Supplementary Figures S7**


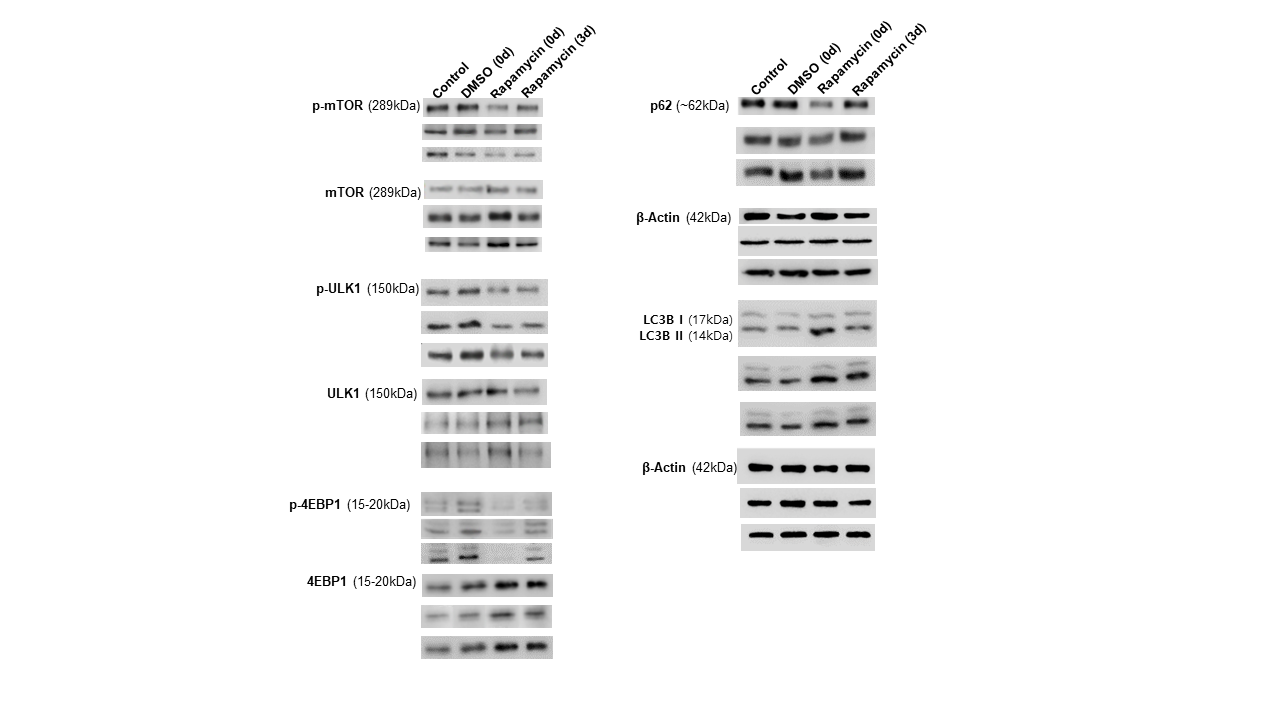


Western blot bands used for analysis for Figure 6.
